# The First Kazakh Whole Genomes: The First Report of NGS Data

**DOI:** 10.5195/cajgh.2014.146

**Published:** 2014-12-12

**Authors:** Ainur Akilzhanova, Ulykbek Kairov, Saule Rakhimova, Askhat Molkenov, Arang Rhie, Jong-Il Kim, Jeong-Sun Seo, Zhaxybay Zhumadilov

**Affiliations:** 1 Center for Life Sciences, Nazarbayev University, Astana, Kazakhstan; 2 Department of Biochemistry and Molecular Biology, Genomic Medicine Institute, Seoul National University College of Medicine, South Korea

**Keywords:** whole genome sequencing, Kazakh population

## Abstract

**Introduction:**

The human genome sequence will underpin human biology and medicine in the next century, providing a single, essential reference to all genetic information. Extraordinary technological advances and decreases in the cost of DNA sequencing have made the possibility of whole genome sequencing (WGS) feasible as a highly accessible test for numerous indications. The international project “Genetic architecture of Kazakh population” is well underway to determine the complete DNA. Next generation sequencing is a powerful tool for genetic analysis, which will enable us to uncover the association of loci at specific sites in the genome associated with disease. The aim of this study was to introduce first data on WGS of 6 Kazakh individuals.

**Methods:**

This pilot study is among the first WGS performed on 6 healthy Kazakh individuals, using next generation sequencing platform HiSeq2000, Illumina by manufacturer’s protocols. All generated *.bcl files were simultaneously converted and demultiplexed using bcl2fasta application. Alignment of sequence reads performed using bwa-mem against human b19 reference genome. Sorting, removing of intermediate files, *.bam files assembling, and marking duplicates were performed using PicardTools package. GATK haplotype caller tool was used for variant calling. ClinVar, SNPedia, and Cosmic databases were processed to identify clinical genomic variants in 6 Kazakh whole genomes. Java Runtime Environment and R. Bioconductor packages were installed to perform raw data processing and run program scripts.

**Results:**

The sequence alignment and mapping procedures on reference genome hg19 of each 6 healthy Kazakh individual were completed. Between 87,308,581,400 and 107,526,741,301 total base pairs were sequenced with average coverage x29.85. Between 98.85% and 99.58% base pairs were totally mapped and on average 96.07% were properly paired. Het/Hom and Ti/Tv ratios for each whole genome ranged from 1.35 to 1.52 and from 2.07 to 2.08, respectively. We compared and analyzed each genome with on existing clinical databases ClinVar, SNPedia, Cosmic and found from 20 to 25, from 269 to 288, from 7 to 12 SNP records, respectively. The availability of a reference Kazakh genome sequences provides the basis for studying the nature of sequence variation, particularly single nucleotide polymorphisms.

**Conclusion:**

The first whole genome sequencing of Kazakhs were performed. In this pilot study, we identified SNPs associated with different conditions. Further studies of WGS on Kazakh population are needed to identify possible unique genetic variants in Kazakhs.

